# Soft matter mechanics of immune cell aggregates

**DOI:** 10.1098/rsif.2025.0231

**Published:** 2025-07-23

**Authors:** Shohreh Askari, Guillem Saldo Rubio, Anagha Datar, Heidi Harjunpää, Susanna C. Fagerholm, Matilda Backholm

**Affiliations:** ^1^ Department of Applied Physics, Aalto University, Espoo, Finland; ^2^ Molecular and Integrative Biosciences Research Programme, Faculty of Biological and Environmental Sciences, University of Helsinki, Helsinki, Finland

**Keywords:** immune cells, cellular aggregates, immune cell activation, soft matter mechanics

## Abstract

T-cells are a crucial subset of white blood cells that play a central role in the immune system. When T-cells bind antigens, it leads to cell activation and the induction of an immune response. If T-cells are activated by antigens *in vivo* or artificially *in vitro,* they form multicellular aggregates. The mechanical properties of such clusters provide valuable information on different T-cell activation pathways. Furthermore, the aggregate mechanics capture how T-cells are affected by mechanical forces and interact within larger conglomerates, such as lymph nodes and tumours. However, an understanding of collective T-cell adhesion and mechanics following cell activation is currently lacking. Probing the mechanics of fragile and microscopically small living samples is experimentally challenging. Here, the micropipette force sensor technique was used to stretch T-cell aggregates and directly measure their Young’s modulus and ultimate tensile strength. A mechanistic model was developed to correlate how the stiffness of the mesoscale multicellular aggregate emerges from the mechanical response of the individual microscopic cells within the cluster. We show how the aggregate elasticity is affected by different activators and relate this to different activation pathways in the cells. Our soft matter mechanics study of multicellular T-cell aggregates contributes to our understanding of the biology behind immune cell activation.

## 1. Introduction

Cells are the fundamental building blocks of life. In multicellular organisms, individual cells work collectively to maintain the structural and functional integrity of tissues and organs. To study these multicellular living materials with the rigour of biological physics and mathematics, multicellular aggregates have been proposed as the ideal model system [1]. Decoding how cells behave and interact in an aggregate is key to understanding diverse biological processes, from tissue regeneration [2] and morphogenesis [3], to immune responses [4] and the progression of diseases like cancer [5].

Microscale force probes, such as atomic force microscopy, optical tweezers and micropipette-based techniques, have been widely used to measure the mechanical properties of single cells [6–13] as well as their adhesion to surfaces or other cells [14–23]. In addition, non-direct force measurements have been performed using, for example, the centrifugation assay, spinning disc and flow chamber, to probe the adhesion force of populations of single cells [24]. However, probing the biomechanics and dynamics of multicellular aggregates is challenging due to the mesoscale length scale of the system. This has led to the development of several new force probes and experimental approaches. For example, Foty *et al.* used a parallel plate compression apparatus to probe the interfacial tension of liver cell aggregates [25]. This approach was later adapted by several others [26,27]. Kalantarian *et al.* used centrifugation to measure the surface tension of aggregates [28]. Ryan *et al.* probed the spreading rates of fibroblast cell aggregates to probe their cell–cell cohesion [29] and the group of Brochard–Wyart has further studied the spreading and flow of cellular aggregates [30–32]. In the same group, Guevorkian *et al.* also developed the micropipette aspiration technique to directly measure the viscoelastic mechanical properties of cancer cell aggregates [33] as well as the mechanosensitive ‘shivering’ of the aggregates under controlled aspiration [34]. Furthermore, Gonzalez-Rodriguez *et al.* measured the detachment and fracture of cellular aggregates using a force-calibrated glass slide as a cantilever [35]. As a final example, Lyu *et al.* have used a soft resistive force-sensing diaphragm to probe the tiny contractile force of cardiac organoids [36]. In general, experimental soft matter physics has been instrumental in forming a new understanding of these living multicellular materials.

From a soft matter mechanics perspective, T-cells are a particularly interesting cell type. These highly specialized, microscopically small immune cells are crucial in the adaptive response of the immune system of mammals [37]. T-cells originate in the bone marrow and migrate to the thymus for further development and maturation. The mature T-cells circulate in the blood stream, encounter antigens in lymph nodes, become activated and finally migrate to peripheral tissues to participate in the intricate functions of the whole immune system. Their main task is to recognize and destroy foreign invaders such as bacteria and viruses. They do this by recognizing and binding to a specific structure called an antigen on, for example, bacteria. Seminal biophysical studies have shown interesting mechanisms of how T-cells use mechanical forces in their immunological response [38,39].

**Figure 1.**
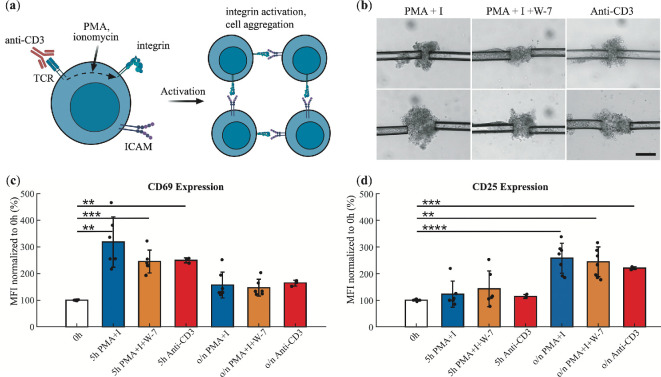
*In vitro* activation of T-cells. (a) Overview of *in vitro* activators: anti-CD3 binds the T-cell receptors (TCRs) and triggers the downstream activation signalling. PMA and ionomycin together mimic part of this signalling cascade. W-7, a calmodulin inhibitor, interferes with some effects of calcium influx into T-cells. T-cell activation leads to integrin activation, integrin binding to intercellular adhesion molecules (ICAMs), and cellular aggregation. It also induces various other cellular changes, including the expression of T-cell activation markers (not shown). (b) Optical microscopy images of T-cell aggregates (held by two micropipettes) following activation with PMA and ionomycin (PMA+I, left); PMA, ionomycin, and W-7 (PMA+I + W-7, middle); and anti-CD3 (right). Scale bar 50 μm. (c) Expression levels of the early T-cell activation marker CD69 after stimulation with PMA+ionomycin, PMA+ionomycin + W-7, or anti-CD3 at 5 h post-induction or overnight (o/n). Presented as mean fluorescence intensity (MFI) as measured using a flow cytometer. (d) Expression levels of the late T-cell activation marker CD25 under the same conditions. The stars in (c)–(d) indicate statistical significance of Welch’s *t*‐test of the mean of a pair of groups with **p < 0.01, ***p < 0.001, ****p < 0.0001.

Upon encountering a specific antigen, T-cells undergo activation and clonal expansion, that is, a rapid division to produce many identical cells that can respond to the same antigen [37]. To study the biology of T-cell activation, the activation process can be mimicked in an antigen-agnostic manner. This means that the T-cells are activated in a way that does not depend on recognizing a specific antigen but still triggers their activation and expansion. Such artificial activation can be done using chemicals such as phorbol myristate acetate (PMA) and ionomycin [40], or anti-CD3 antibodies [4,22]. These different activators work through distinct mechanisms (figure 1a). Anti-CD3 binds directly to the T-cell receptor (TCR), mimicking antigen binding and triggering a signalling cascade that results in T-cell activation. PMA and ionomycin stimulate elements of the intracellular signalling pathway that follow antigen recognition. Another important compound for the T-cell activation pathway is the inhibitor W-7, which inhibits the function of calmodulin (Ca^2+^-binding protein) in cells [41].

When T-cells are activated, this leads to integrin activation and T-cell clustering into large aggregates of many cells (figure 1b). These aggregates are biologically important, as they allow the cells to efficiently share important soluble factors (interleukin-2) that allow for cell proliferation [42]. Integrins play a vital role in the immune cell function, particularly in T-cell adhesion, mediated by interactions with intercellular adhesion molecules (ICAMs) [21]. Atomic force microscopy studies have revealed time-dependent adhesion strengthening, with unbinding forces increasing from 140 to 580 pN over short time scales [21] as well as the effect of force generation on T-cell activation [43]. Advances in optical tweezers have provided further insights into integrin mechanics, enabling the quantification of cell–cell bond rupture forces [22].

These biophysical approaches collectively enhance our understanding of integrin function at molecular and cellular levels. However, an understanding of collective T-cell adhesion and mechanical forces in cell aggregates following cell activation is currently completely lacking. Such information is crucial because immune cells often interact within larger conglomerates, such as lymph nodes and tumours, and mechanical forces between cells are emerging as important regulators of immune cell programming, behaviour and function [44].

Here, we have used a soft matter mechanics approach to take a first step towards understanding the biology of T-cell interactions in mesoscale multicellular aggregates. In our experiments, we have activated mouse primary T-cells to study how the different activation pathways affect the resulting immune cell activity and multicellular aggregate properties. By using the micropipette force sensor (MFS) technique, we have directly measured the mechanical properties of the *in vitro* activated immune cell aggregates through stretching. To correlate the emergent macroscopic mechanical response of the multicellular system with the microscopic cell elasticity, we have developed a new mechanistic model. We have explored the effects of various factors, such as post-induction time, aggregate volume and pre-stretching, on the T-cell aggregate. Finally, we evaluated the influence of specific activators, including W-7 (a calmodulin antagonist) and anti-CD3, on aggregate stiffness. Our results showcase the importance of a multidisciplinary approach in the study of biological systems.

## 2. Methods

In this section, we concisely describe our experimental approach. For all technical details on methodology and materials, refer to §6 at the end of the article.

### 2.1. *In vitro* induced T-cell aggregation

To study the cohesion and mechanics of immune cell aggregates, primary T-cells were isolated from mice and activated using PMA and ionomycin, or by only anti-CD3. When the calmodulin inhibitor W-7 was used, it was added 30 min before the addition of PMA and ionomycin. These chemicals trigger intracellular signalling events that activate integrins, making the T-cells highly adhesive and causing them to aggregate into clusters (figure 1b). As shown in figure 1c,d, these treatments induce T-cell activation, as evidenced by increased CD69 expression at 5 h and CD25 expression after overnight incubation [45]. Of these *in vitro* activators, anti-CD3 most closely mimics the biological activation of T-cells, initiating intracellular signalling within the T-cell. PMA and ionomycin mimic these signalling effects by artificially activating the same pathways as T-cell receptor activation [46]. W-7, a calmodulin antagonist, further modulates T-cell signalling and introduces an additional regulatory dimension to PMA+ionomycin-induced activation [47,48].

### 2.2. Stretching of T-cell aggregates

**Figure 2.**
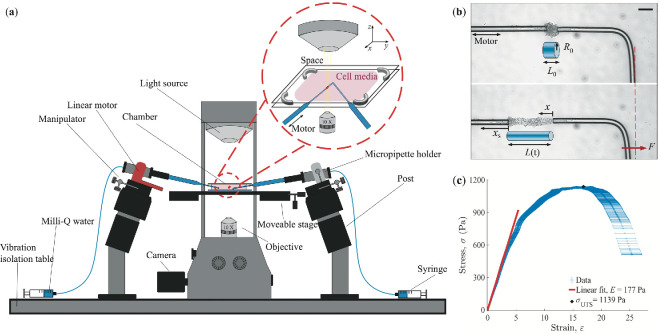
Cell aggregate stretching using the micropipette force sensor. (a) Schematic sketch (not to scale) of the set-up with the straight and L-shaped micropipettes holding on to the T-cell aggregate with suction. The straight micropipette is connected to a linear motor and the L-shaped is calibrated and used as a force sensor. (b) Examples of optical microscopy images from before (top) and after (bottom) the stretching. The aggregate is modelled as a cylinder with an initial radius 𝑅_0_ and length 𝐿_0_. During the experiment, the straight micropipette is moved to the left at a constant speed (𝑥_S_ = 𝑣𝑡), causing the L-shaped force sensor to deflect (𝑥). This applies a Hookean force 𝐹 = 𝑘_p_𝑥 onto the aggregate, which stretches, 𝐿(𝑡). Scale bar 50 μm. (c) Example of stress–strain graph from a typical stretching experiment. The Young’s modulus 𝐸 and ultimate tensile strength 𝜎_UTS_ are determined from the graph. The error bars are error propagations of the stress and strain using the standard deviation of 𝑘_p_, 𝑅_0_ and 𝐿_0_ (see §6 for details).

The MFS technique [49] was used to stretch the T-cell aggregates and measure their mechanical properties (figure 2). In this technique, the deflection x of a glass micropipette is used to measure and apply a force $F=k_{p}x$ , where $k_{p}$ is the spring constant of the cantilever, determined through calibration. The MFS technique has been extensively used to probe the biomechanical properties of single cells [8,9], including T-cells [39], as well as microbial flocs [50,51] and whole organisms [52,53].

The buffer solution containing T-cells was carefully injected between two glass slides, which were mounted in a custom-made holder on an inverted microscope. Two micropipettes were required for the stretching experiments: one straight holding micropipette mounted on a linear motor and one L-shaped, force-calibrated MFS mounted at a 90° angle on a manual *xyz*-micromanipulator (figure 2a). These micropipettes were moved into the chamber, ensuring that the entire lengths of the cantilevers were immersed in the buffer solution. Both micropipettes were used to hold on to an aggregate with gentle suction using syringes. To stretch the aggregate, the straight micropipette was moved to the left at a constant speed of v=20 µm s^−1^ using a linear motor (figure 2b). This movement caused a deflection in the L-shaped micropipette, which applied an increasing force onto the aggregate, leading to its stretching.

## 3. Models

### 3.1. Mechanical properties

The T-cell aggregate was modelled as a cylinder with an initial radius $R_{0}$ and length $L_{0}$ (figure 2b). When stretched using MFS, the aggregate is under a stress $\sigma =F/\pi R_{0}^{2}=k_{p}x/\pi R_{0}^{2}$ and strain $\varepsilon =\Delta L/L_{0}$ , where $\Delta L=x_{S}-x=vt-x$ is the change in length of the aggregate. We have, in other words, used the engineering stress instead of the true stress to determine the mechanical properties. The same choice of definition was used in the cell aggregate stretching work of Gonzalez-Rodriguez *et al.* [35], allowing for direct comparisons between our findings. The Young’s modulus is defined as E=σ/ε for small deformations within the elastic regime [54]. This corresponds to the slope of the initial, linear regime in the standard stress–strain graph, illustrated with an example of our experimental data in figure 2c. As the aggregate is stretched further, the system transitions into the plastic regime, where material deformations become irreversible. From the stress–strain graph, the ultimate tensile strength $\sigma_{UTS}$ was determined (figure 2c), which is an important measure of the mechanical properties of the aggregate, representing the maximum stress the material can sustain before breaking.

### 3.2. Mechanistic model

**Figure 3.**
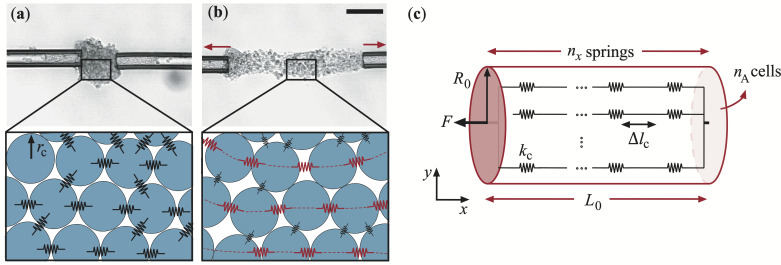
Mechanistic model. The cells are modelled as spheres with a radius of 𝑟_c_ and spring constant 𝑘_c_. The aggregates with their modelled cell-spring structure are shown in (a) before and (b) during the stretching. Scale bar 50 μm. (c) Schematic drawing of the mechanistic model with parallel connections of series-connected springs acting as force chains through the aggregate. Each spring changes its length by Δ𝑙_c_ when the external force 𝐹 is applied.

Various theoretical models, such as the geometry-based vertex model and lattice-based cellular Potts model [1], exist for describing a multicellular system. Most of these focus on epithelial morphology, topology, dynamics and mechanics in two dimensions [3,55–57]. A limited focus has been on modelling three-dimensional systems [57–62]. In our case, an advantage of a vertex model over the cellular Potts model would be that it allows for the treatment of the elastic response of a multicellular system under external loading [1]. However, the vertex-based model is not suitable for describing T-cell aggregates, where the randomly organized small cells do not deform into polyhedral shapes as required in a three-dimensional vertex geometry. To bridge the gap between individual cellular mechanical response and the emergent macroscopic mechanical properties (defined in §3.1), a new scaling law-based mechanistic model was developed (figure 3). A T-cell aggregate was assumed to consists of randomly arranged cells adhered to each other. The mechanical response of the cell (radius $r_{c}$ ) was modelled with an elastic spring with a stiffness of $k_{c}$ . This effective cell stiffness includes the stiffness of the cell–cell adhesion bonds coupled with that of the cell itself, where we assume that the stiffness stems from the elastic cortical shell of the cell. The deformations of these springs contribute to the emergent mechanical response of the aggregate.

The force is applied in the *x*-direction, causing a mechanical response of the cells mostly along this axis. In response, each spring extends by $\Delta l_{c}$ , forming a force chain in the aggregate, where neighbouring cells along the *x*-axis create a series connection of springs (figure 3b,c). For one such series connection along the entire aggregate length, the number of springs is $n_{x}∼L_{0}/2r_{c}$ . To stretch one such series-connected chain of springs, a force of $F_{series}=k_{c}\Delta l_{c}$ would be required [63]. The change in length of an entire chain upon stretching is the same as the change of length of the entire aggregate: $\Delta L∼n_{x}\Delta l_{c}∼{(L}_{0}/2r_{c})\Delta l_{c}$ .

The total number of series-connected chains within the cylindrical cross-sectional area ( $A=\pi R_{0}^{2}$ ) of the aggregate is $n_{A}∼R_{0}^{2}/r_{c}^{2}$ . These chains of springs respond in parallel when the entire aggregate is stretched (figure 3c), requiring a total force of $F=n_{A}F_{series}$ , according to the conventional rules of stretching parallel connected springs [64]. This gives an expression for the total force needed to stretch the aggregate: $F={k_{c}\Delta l_{c}R}_{0}^{2}/r_{c}^{2}$ .

Adding all of this together, the Young’s modulus can thus be written as


(3.1) $$E=\frac{\sigma }{\varepsilon }=\frac{F}{A}\frac{L_{0}}{\Delta L}∼\frac{k_{c}}{r_{c}}.$$

This equation links the emergent mechanical response of the entire multicellular aggregate to the stiffness of the individual cells and their internal bonds. It shows that the elasticity of the aggregate scales linearly with the elasticity of the cells. This finding is critical for understanding the relationship between T-cell activation levels and aggregate mechanics, as higher T-cell activation can be hypothesized to create stronger adhesion within the aggregate as well as affect the cell stiffness. It is important to note that this mechanistic model is only valid in the initial elastic regime and cannot be extrapolated to the later stages of the stretching experiments.

## 4. Results and discussion

### 4.1. Mechanics of T-cell aggregates

**Figure 4.**
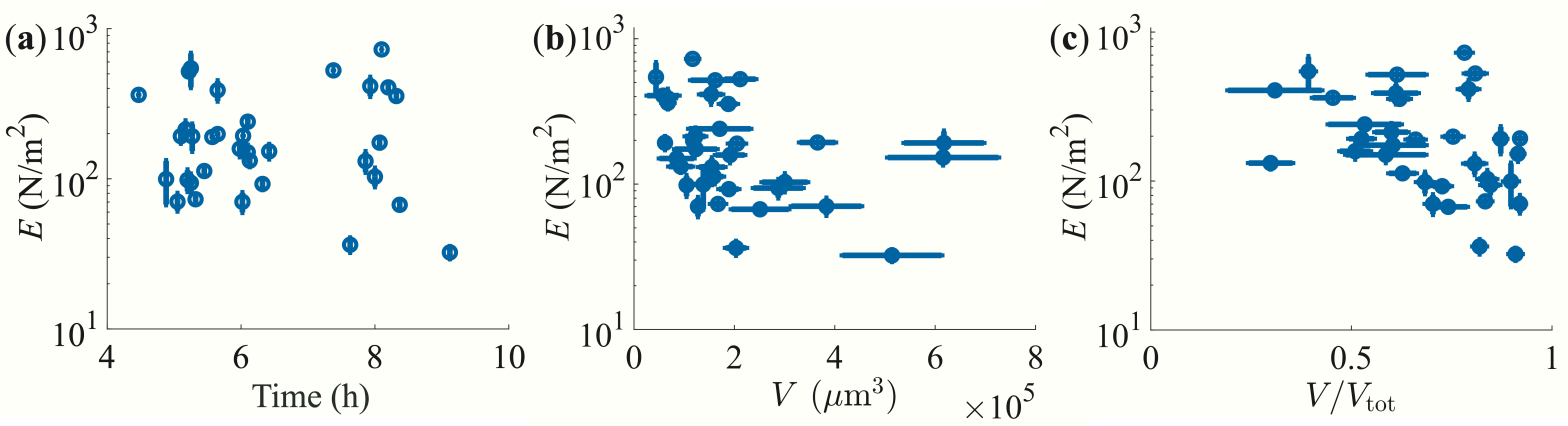
Effect of time, volume and aspiration. Young’s modulus of T-cell aggregates (activated with PMA and ionomycin) as a function of (a) time after activation, (b) cluster volume and (c) relative cluster volume outside of the micropipettes. Within error, the stiffness remains constant for all cases.

T-cell aggregates formed through activation with PMA and ionomycin were used as a model system in this study. The cells were activated in a biology laboratory and the aggregates were then transported to a physics laboratory for measurements. The activated T-cells remained viable for at least 24 h post-induction. A representative example of an MFS stretching experiment is shown in electronic supplementary material, video S1 and its resulting stress–strain curve is shown in figure 2c. The MFS technique is well suited for these measurements because of its ability to precisely measure nN-range forces while simultaneously stretching the sample and observing the resulting deformation. Since the sample is held through gentle suction with the micropipettes, no potentially harmful glue is needed. Several aggregates of varying volumes ( ${V=10}^{4}$ to ${7\times 10}^{5}$ µm^3^) were stretched (see electronic supplementary material, videos S2–S3). All experiments showed very similar stress–strain graphs with the clear traits of ductile material, indicating significant plastic deformations before rupture. In figure 4a, the Young’s modulus is plotted as a function of time from when the activation was done. There is no effect of post-activation time on the stiffness of the clusters—as long as the cells remain viable, the Young’s modulus remains constant.

Within error, the measured mechanical properties of the aggregates were independent of their volume (figure 4b). Experiments were initially also performed on aggregates with volumes much larger than 10^6^ µm^3^. However, these were found to be more irregular in shape with portions of the cluster not contributing to the stretching process. Since the cylindrical shape approximation did not properly account for the actual stretching of the large aggregates, these clusters were discarded from our results.

To hold on to the aggregate during the stretching experiment, a fraction of it was aspirated into both micropipettes, as seen clearly in the optical microscopy images of figure 3. In figure 4c, the measured Young’s modulus is plotted as a function of the volume V of the aggregate (outside of the micropipettes) divided by the total volume $V_{tot}$ of the aggregate (inside and outside of the micropipettes). Within experimental error, there is no mechanobiological effect of the aspiration on the measured stiffness.

**Figure 5.**
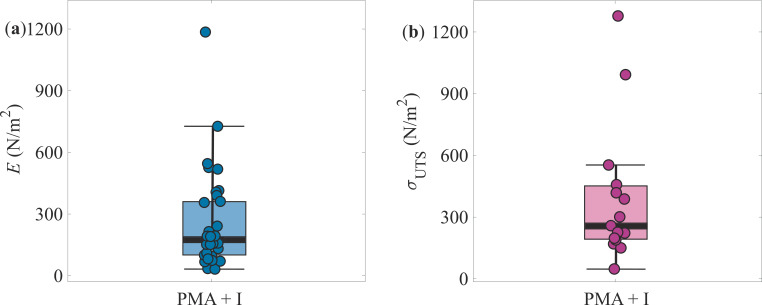
Mechanical properties. (a) The Young’s modulus and (b) ultimate tensile strength of T-cell aggregates activated with PMA and ionomycin (i). The thick black line inside the box represents the median. The box spans the interquartile range (IQR), covering the middle 50% of the data. The error bars (whiskers) extend to the minimum and maximum values within 1.5 times the IQR, while points beyond this range are considered outliers.

The Young’s modulus and ultimate tensile strength data are summarized in figure 5. The average values across 35 aggregates were determined as E=248±234 Pa and $\sigma_{UTS}=390\pm 334$ Pa (median values shown with black lines in figure 5). In comparison, the stiffness of *individual*, non-activated T-cells has been measured as 80−100 Pa [65,66], whereas activated T-cells have stiffnesses in the range of 100−300 Pa [39]. The increase in stiffness during activation is due to drastic modifications of the actomyosin cytoskeleton. The stiffness of the aggregates is related mainly to changes in the actin cytoskeleton at the contact sites between the cells. Upon cell activation (through the T-cell receptor or with phorbol ester and ionomycin), the cells cluster together with the help of cell adhesion receptors, which leads to downstream signalling events. Integrin–actin linkages, actin polymerization and actin cross-linking (and later on centrosome and Golgi polarization) occurs at the contact sites between the cells, allowing for stiffening of the aggregates.

Our measured mechanical properties are lower than those reported for murine sarcoma aggregates [33,35]. Specifically, micropipette aspiration experiments on these yielded a Young’s modulus of E=700±100 Pa [33]. Measurements using glass slide-based cantilevers showed Young’s modulus values ranging from E=1000±300 Pa at very low speeds (*v* < 0.02 µm s^−1^) to E=9000±2000 Pa at higher speeds (*v* = 1 to 50 µm s^−1^) [35]. Our T-cell aggregates may be softer due to the big differences in adhesion mechanisms and cytoskeletal architecture between immune cells and sarcoma cells. The latter study reported maximum stress values of $\sigma_{max}=$ 300–700 Pa, which are similar, within error, to our measurements.

By combining the measured elastic modulus with our mechanistic model (equation (3.1)), the elastic response of one cell (including both the stretching of the cell as well as the deformation of the cell–cell bonds) $k_{c}∼r_{c}E$ can be determined. The average T-cell radius was measured as $r_{c}=3.3\pm 0.8$ µm, giving an average cell spring constant of $k_{c}\approx 8\times 10^{-4}$ N m^−1^. This spring constant agrees well with the results in Lek *et al.* [21], where atomic force microscopy was used to measure the adhesion force between a single effector T-cell and an integrin ligand-coated surface. From their data, a spring constant of approximately $10^{-4}$ N m^−1^ was extracted, which captures both the deformation of the cell as well as the cell–cell adhesion bonds. This agreement underscores the reliability of our experimental and theoretical approach in quantifying T-cell mechanics within cellular aggregates.

In the mechanistic model, we assumed a purely elastic system and that the effect of the lateral bonds between the cells is much smaller than that in the direction of stretching. In the future, a more refined model could be developed to capture how the cells are interconnected. To reliably test such a new model, more advanced stretching experiments using confocal or fluorescence microscopy would be required to track the position and deformation of each cell during stretching as well as their connection points to nearest neighbours. Here, we only used one stretching speed ( v=20 µm s^−1^) to deform the aggregates and probe the elastic response of these. This was to ensure a high enough number of successful experiments given the large variations in the biological sample. In future work, it would be interesting to perform the experiments at different stretching speeds to probe the viscoelastic response of the multicellular material. A more advanced mechanistic model, incorporating an elastic cell shell and its viscoelastic interior, would then be needed to describe the mechanical response.

Stretching experiments were also performed on cells containing a mutation in the β2-integrin that affects T-cell adhesion under flow conditions [67]. These knock-in cells were activated with PMA and ionomycin, but no difference was detected between the knock-in and the wild-type cellular aggregates (see electronic supplementary material). This indicates that the integrin mutation is not important for regulating cell–cell aggregate properties or activation under these activation conditions, as also shown in our previous work [67].

### 4.2. Pre-stretching of T-cell aggregates

**Figure 6.**
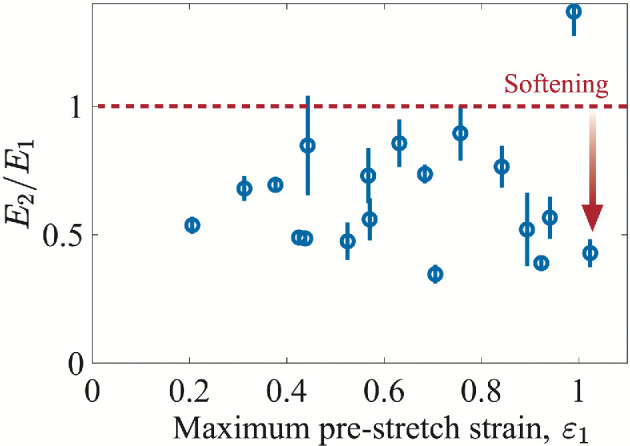
Effect of pre-stretching. The ratio between the Young’s modulus in the second (𝐸_2_) and first (𝐸_1_) stretching experiment as a function of maximum strain 𝜀_1_ during the pre-stretching. The aggregates soften after the pre-stretching.

Cells respond to mechanical stimuli by altering their properties. To investigate this in our aggregates, double-stretching experiments were performed. The aggregates were first pre-stretched to a maximum strain of $\;\varepsilon_{1}\approx$ 0.2–1, then returned to their initial configurations before being stretched again until rupture (see example experiment in electronic supplementary material, video S4). The average total waiting time between the successive stretches was 9.3±2.1 s and was kept constant for all experiments. Some relaxation of the aggregate occurred during this time. The Young’s modulus was measured in both elastic stretching phases, and the change in stiffness ( $E_{2}/E_{1}$ , where 1 and 2 refer to the first and second stretch, respectively) is plotted as a function of $\varepsilon_{1}$ in figure 6.

Our results show that aggregates soften in response to pre-stretching by a factor of 0.7±0.3 . This is in contrast to previous findings, where mechanical stimulation led to stiffening due to integrin–ICAM catch bond formation [68]. Instead, the viscoelastic relaxation of the aggregates during and after the first stretching phase is likely to have relaxed the aggregate modulus. The pre-stretch could have caused a disruption of the actin cytoskeleton, also known as ‘fluidization’ [69], which has been shown to occur at higher stretch amplitudes or rates, without affecting cell adhesion. Fluidization of the actin cytoskeleton is due to a broad effect on the whole cytoskeleton rather than a single molecular/signalling event.

### 4.3. Effect of T-cell activation pathway

**Figure 7.**
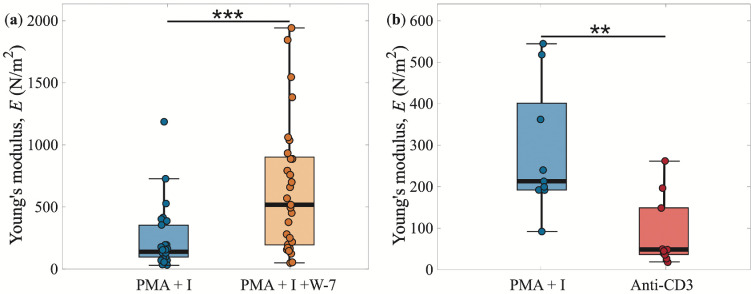
Activation pathways. The Young’s modulus of T-cell aggregates activated with PMA+ionomycin compared with (a) aggregates activated also with W-7 and (b) only with anti-CD3. The addition of W-7 makes the aggregates stiffer, whereas anti-CD3 produces softer aggregates than PMA+ionomycin. The stars indicate **p < 0.01 and ***p < 0.001.

When T-cells were treated with W-7 in addition to the standard PMA and ionomycin, the aggregates exhibited increased stiffness compared with those activated with only PMA and ionomycin (figure 7a, see example experiment in electronic supplementary material, video S5). This effect was more pronounced when comparing data from experiments conducted on the same day, using cells from the same mouse. Specifically, the addition of W-7 increased aggregate stiffness by a factor of $E_{PMA+I+W7}/E_{PMA+I}=2.8\pm 0.7$ (based on same-mouse experiments). This increase directly translates to a stiffening of the cells including the cell–cell adhesion bonds) by the same factor (equation (3.1)). Biologically, this suggests that T-cells become stiffer in response to W-7, probably due to its inhibition of Ca^2+^-calmodulin, which itself is an inhibitor of caldesmon–actin bundle binding. By this mechanism, W-7 may be responsible for enhancing T-cell stiffness [70]. Interestingly, we also detected a small (not statistically significant) increase in metabolic activity in W-7-treated cells (see electronic supplementary material, [71]), indicating that altered mechanical responses of the aggregates may also be reflected in the activation response of the cells, for example altered cell proliferation, which we have not explored here.

To further explore the effect of different activation pathways, T-cells were activated using either anti-CD3 antibodies or PMA and ionomycin. Anti-CD3 antibodies provide a more physiologically relevant activation method, as they induce the same conformational change in CD3 (the T-cell antigen receptor) that occurs when it recognizes an antigen *in vivo*. This conformational change initiates the intracellular signalling cascade. The activation with anti-CD3 rendered a clear decrease in the aggregate stiffness as compared with cells activated with PMA and ionomycin (figure 7b, see example experiment in electronic supplementary material, video S6). Comparing the change in stiffness for aggregates with cells from the same mouse, $E_{Anti-CD3}/E_{PMA+I}=0.30\pm 0.02$ . This suggests a lower level of activation of the T-cells with the antibody as compared with using the more artificial chemicals (which cause maximal activation of the cells). This correlates with lower CD69 expression in anti-CD3 treated cells compared with PMA/ionomycin activation, e.g. lower level of activation (figure 1c). Anti-CD3 is known to stiffen individual T-cells by a factor of two upon activation [39]. Our results indicate that PMA+ionomycin either stiffen the cells by a factor of *ca* 4 upon activation, or that the aggregate stiffness is primarily determined by cell–cell adhesion strength rather than individual cell elasticity.

The immune system is a complex cellular system that mediates functions such as protection against infection and tumour surveillance, but is also involved in pathological events, for example autoimmunity. To mediate these functions, immune cells interact both with other immune cells and non-immune cells, forming interconnected cellular communities. The human immune system consists of approximately 1.8 trillion cells, with a total weight of 1.2 kg. Of these, up to 40% are lymphocytes (T-cells and B-cells) [72]. These cellular communities interact in a highly coordinated manner for the immune system to perform its functions. However, lymphocytes are small and motile and mediate transient interactions with other immune cells and non-immune cells, making their analysis more challenging than the stable interactions found in other tissues and organs in the body.

Transient interactions between immune cells are mediated by cell surface proteins, and their complexity is beginning to be unravelled [73,74]. Immune cells communicate with each other through these cell surface receptors, which initiate intracellular signalling events within the cells, leading to cellular outcomes such as activation and effector functions. The intracellular signals consist of biochemical signals, but more recently, it has become apparent that also mechanical signals are important to determine outcome in immune reactions, such as lymphocyte activation [75]. Indeed, increased stiffness of the environment leads to stronger T-cell activation [76,77]. However, mechanical forces in larger immune cell communities have so far remained uncharacterized due to technical challenges to measure such forces in the small and transient cell groups. Here, we have made a first attempt to characterize mechanical properties of immune cell communities, starting with small T lymphocyte aggregates, and to correlate these with cellular activation outcomes by using novel methodology.

## 5. Conclusions

In this work, the micropipette force sensor technique was used to directly measure the mechanical properties of mesoscale (diameter approx. 30–120 µm) T-cell aggregates through stretching experiments. The multicellular aggregates were formed upon *in vitro* activation using PMA and ionomycin and their Young’s modulus and ultimate tensile strength were determined as E=248±234 N m^−2^ and $\sigma_{UTS}=390\pm 334$ N m^−2^. These values are similar to the mechanical properties reported in the literature for aggregates made of other cell types. A mechanistic model was developed to couple the emergent mechanical response of the aggregate (Young’s modulus *E*) with the intrinsic mechanical response of the cells (spring constant $k_{c}$ ): $E{∼k}_{c}/r_{c}$ , where $r_{c}$ is the average cell radius. This rendered $k_{c}\approx 8\times 10^{-4}$ N m^−1^ which agrees well with literature values from atomic force microscopy measurements on single T-cells. The addition of W-7 rendered stiffening of the aggregates by a factor of 2.8±0.7 , probably due to its inhibition of Ca^2+^-calmodulin. Conversely, T-cell activation with anti-CD3 rendered softening of the clusters by a factor of 0.30±0.02 compared with aggregates activated with PMA and ionomycin. This suggests a lower level of activation of the T-cells with the antibody as compared with using the more artificial chemicals, which cause stronger activation of the cells.

## 6. Experimental details

### 6.1. T-cell isolation

The day before an extraction the MACS buffer was prepared following Miltenyi’s instructions: phosphate buffered saline (PBS, Lonza, cat. no. 17-516F), 0.5% bovine serum albumin (BSA, Biowest, cat. no. P6154) and 2 mM ethylenediaminetetraacetic acid (EDTA). After the BSA was fully dissolved, the solution was sterilized through filtration through a 22 µm nylon mesh filter (Fisher Scientific, cat. no. 2263548).

Spleens from C57BL/6 N mice were extracted and mechanically dissociated by grinding the tissue between the back of a sterile syringe and a 70 µm cell strainer (Fisher Scientific, cat. no. 22363548) placed over a 50 ml falcon after the cell strainer was wetted using 1 ml of ice-cold PBS (Lonza, cat. no. 17-516F) supplemented with 2% fetal bovine serum (FBS, Gibco, cat no. 10500-064). After the tissue was dissociated, the syringe back and the strainer were rinsed using a further 5 ml of ice-cold PBS + 2% FBS. The cell strainer was discarded, and the cell suspension was centrifuged at 330*g* for 5 min. The supernatant was discarded, and the pellet was resuspended in room temperature ammonium-chloride-potassium (ACK) lysis buffer (0.15 mol l^−1^ NH_4_Cl, 0.01 mol l^−1^ KHCO_3_ and 0.1 mmol l^−1^ EDTA in milli-Q water) to perform red blood cell lysis. With our aliquot the time needed to do so was 50 s. After this time the cell suspension was diluted to a final volume of 20 ml with PBS + 2% FBS. The cell suspension was passed through a 70 µm cell strainer and into a new falcon. The suspension was then centrifuged at 330*g* for 5 min and the supernatant discarded. The pellet was resuspended in 40 µl of de-gassed MACS buffer + 10 µl of the biotinylated antibody cocktail from the CD4+T cell enrichment kit (Miltenyi, cat. no. 130-104-454) and placed on ice for 5 min. After the initial incubation on ice, 30 µl of MACS buffer and 20 µl of the anti-biotin magnetic beads from the same kit were added, mixed well and incubated on ice for 10 min.

During the incubation, the magnetic separation column (Miltenyi, cat. no. 130-042-401) was placed on the magnet over a 15 ml falcon and capped with a 70 µm cell strainer and primed by adding 3 ml of MACS buffer. After the 10 min incubation of the magnetic beads was done, the cell suspension and beads were resuspended in 1 ml of MACS buffer and transferred to the magnetic separation column. After the cell suspension flows through the column, the falcon is rinsed with 1 ml of MACS buffer and the liquid was added to the column. After this, the column was washed with more MACS buffer until 10 ml of MACS buffer and CD4+T cell enriched cell suspension made it through the column. The cell suspension was then counted and centrifuged at 330*g* for 5 min. The supernatant was discarded, and the cell pellet was resuspended in RPMI (Lonza, cat. no. 12-167F/EuroClone, cat. no. ECB9006L) supplemented with 10% FBS (Gibco, cat no. 10500-064), 100  U ml^−1^ penicillin–streptomycin (penicillin, Orion, cat. no. 465161; streptomycin Thermo Fisher Scientific, cat. no. D7253-100 g), 2  mM L-glutamine (Thermo Fisher Scientific, cat. no. BP379-100) and 50  µM 2-mercaptoethanol (Fluka biochemika, cat. no. 63690) at a concentration of 4 million cells ml^−1^ and plated in a 24-well plate (CellStar, cat. no. 662160) and incubated at 37°C before proceeding to the different experiments.

### 6.2. T-cell treatments

The T-cell suspension was activated using anti-CD3 2.5 µg ml^−1^ (Sigma-Aldrich, cat. no. MAB484) or PMA 10 ng ml^−1^ (Sigma-Aldrich, cat. no. P81389) + Ionomycin 0.5 µg ml^−1^ (Thermo Fisher Scientific, cat. no. J62448.M). In the cases where the calmodulin inhibitor W-7 (Sigma-Aldrich, cat. no. 681629) was used, it was added at 50 µM 30 min before the addition of PMA + Ionomycin.

### 6.3. Flow cytometry

The following conjugated antibodies were used for flow cytometric analysis of Puromycin-PE (BioLegend, cat. no. 381503, clone 2A4): CD4-PE-Cy7 (BioLegend, cat. no. 100528, clone RM4-5), CD25-APC-Cy7 (BioLegend, cat. no. 102026, clone PC61), CD69-APC (BioLegend, cat. no. 104514), clone H1.2F3. Fc-receptor block (BD Pharmingen, cat. no. 553142 clone 2.4G2) was used in all experiments assessing mouse cells, and unstained and fluorescence minus one (FMO) controls were included in all panels. 7-AAD (eBioscience, cat. no. 00-6993-50) was used to detect dead cells. Acquisition was performed on an LSR Fortessa flow cytometer (Becton Dickinson), and data were analysed using FlowJo software (Tree Star).

### 6.4. Translation rate analysis

Puromycin (Gibco, cat. no. A11138-03) is incorporated into nascent proteins in a manner that prevents the further extension of said proteins and assayed through flow cytometry using an anti-Puromycin PE conjugated antibody (BioLegend, cat. no. 381503) after fixing and permeabilizing the cells using the Foxp3 permeabilization kit (eBiocience, cat. no. 00-5523-00). To determine the total background for puro-PE fluorescence we used Harringtonine (Sigma-Aldrich, cat. no. SML1091-10MG) at 2 µg ml^−1^ for 30 min before the addition of Puromycin. To determine the maximum energy level of the cells, dimethyl sulfoxide (DMSO, Fisher Scientific, cat. no. BP231-1) 0.08% was used as a negative control and added for 30 min before the addition of Puromycin. To determine the contribution of mitochondrial energy pathways to the total, the mitochondrial ATP synthase inhibitor Oligomycin (Sigma-Aldrich, cat. no. 04876-5MG) was used at 1 µM for 30 min before the addition of Puromycin. To determine the contribution of glucose-related energy production to the total, the phosphoglucoisomerase inhibitor 2-Deoxy-D-Glucose (Sigma-Aldrich, cat. no. D8375-5G) was used for 30 min before the addition of Puromycin. To determine the contribution of glycolysis alone to the cell’s energy level a combination of Oligomycin and 2-Deoxy-D-Glucose can be used. In all cases, Puromycin was added at 10 µg ml^−1^ for 45 min after 30 min of incubation with the different inhibitors. The incorporation of puromycin was then assayed via flow cytometry.

### 6.5. Micropipette force sensor

The MFS was manufactured and calibrated as described in detail in [49]. Briefly, the MFS was made of a hollow glass capillary (World Precision Instruments, TW100-6) with an inner diameter of 0.75 mm and an outer diameter of 1 mm. The cantilever was pulled using a micropipette puller (Narishige, PN-31) and shaped with a microforge (Narishige, MF-900).

Prior to use, the MFS requires calibration to determine its spring constant, $k_{p}$ . We calibrate our cantilevers using the water droplet method developed by Colbert *et al.* [9] and described in detail in [49], using water drops of different sizes as control weights. The cantilever was mounted horizontally, and a droplet of Milli-Q water (Millipore Direct-Q 3 UV) was pushed out to rest on the cantilever end using a syringe connected with a plastic tube to the micropipette holder (IM-H3 Injection Holder Set by Narishige Lifemed Co., Ltd.). A camera was used to image the experiment from the side at a frame rate of 30 frames s^−1^. Using an in-house MATLAB (MathWorks) image analysis script, the shape of the drop was modelled as an ellipsoid with a volume of V . The drop weight was then calculated as W=ρgV , where ρ is the density of water. The cantilever deflection Δx was measured as a function of time through image analysis in MATLAB, and the micropipette spring constant was determined using Hooke’s law: $k_{p}=W/\Delta x$ . The calibration was repeated several times for each cantilever. In this study, micropipettes with spring constants ranging from 10 to 40 nN µm^−1^ were used.

### 6.6. Stretching experiments

#### 6.6.1. Microscope set-up

An inverted wide-field microscope (Nikon ECLIPSE Ts2-FL) equipped with a manually adjustable *xy*-stage was used to securely position the sample chamber. A 10× objective (Nikon 10×/0.25 Ph1 DL Objective Lens) was used to ensure sufficient resolution as well as a big enough field of view to observe the cluster deformation and micropipette motion. Image acquisition was performed using a FLIR camera (GS3-U3-23S6M-C, Integrated Imaging Solutions, Inc.) mounted on the microscope. An image sequence of the experiment was recorded at 30 frames s^−1^, a frame rate chosen to match the experimental speed and ensure adequate temporal resolution.

#### 6.6.2. Experimental chamber

The sample chamber was constructed using aluminium and designed to hold two untreated glass slides at a fixed spacing. Depending on the volume of the cell solution media, two sizes of glass slides were used (Plain Pre-Cleaned Microscope Slides, 75 × 50 mm, thickness of 0.96 to 1.06 mm, and VWR Ground Edge Microscope Slides, 75 × 25 mm, thickness of 0.8 to 1.2 mm). Spacing between the slides was maintained using four plastic spacers, each 2 mm thick, placed at the corners of the slides to create a uniform 2 mm gap (figure 2a). The cell culture media was injected into the chamber using a plastic pipette, with the tip widened by cutting it with scissors to minimize disruption of the clusters during injection. Surface tension prevented leakage from the chamber, while strong intercellular bonds ensured the integrity of the clusters during their transfer from a 24-well plate to the sample chamber.

#### 6.6.3. Micropipette set-up

Cantilevers of two micropipettes were positioned parallel to the *xy*-plane, situated between the glass slides and submerged in the cell culture medium. Each micropipette was connected to a micropipette injection holder (IM-H3 Injection Holder Set by Narishige Lifemed Co., Ltd.), which was linked via plastic tubing (i.d. = 1 mm; Narishige, model no. CT-1) to syringes (sizes ranging from 10 to 20 ml) filled with water. The holders were securely mounted on optical bases (Dynamically Damped Post, 14’ Long, Metric, Ø1.5’, Thorlabs, model no. DP14A/M) fixed to an active vibration isolation stage (MVIS 30 × 30 model by Newport Corporation), minimizing interference from environmental vibrations or nearby movement. Suction pressure, controlled via the syringes, was used to enable the micropipettes to firmly grasp the clusters.

A straight micropipette was attached to a linear motor (Thorlabs Kinesis Brushed Motor Controller) to enable controlled motion along the *x*-axis, with an initial acceleration of 1 mm s^−2^ and a constant velocity of 20 µm s^−1^. An L-shaped micropipette was positioned such that the bent portion of its cantilever was parallel to the cantilever of the straight micropipette. The L-shaped micropipette remained stationary throughout the experiment and functioned as a force sensor.

#### 6.6.4. Experimental procedure

T-cells were extracted and activated at the University of Helsinki and moved to Aalto University for mechanical analysis in 6-well cell culture plates in a Styrofoam box at 37°C. All samples were analysed within 12 h of activation. The prepared cell media contained a high density of single T-cells and a small number of aggregates. Since the cells and aggregates were denser than the surrounding medium, they settled onto the bottom slide of the experimental chamber. To allow for proper settling, the sample was left undisturbed for a few minutes before beginning the experiment. Once an aggregate had stabilized on the bottom slide, the straight micropipette was used to lift it from the surface and position it between the slides. Both micropipettes then grasped the aggregate, ensuring that neither of them made contact with the slides. The linear motor was activated, causing the straight micropipette to move backward and stretch the cluster. This induced deflection in the stationary L-shaped micropipette, which served as a force sensor. The applied force and cluster deformation were determined by analysing the recorded images using MATLAB.

In the cell culture medium, a limited number of aggregates were present, exhibiting variations in size and shape. For the experiments, aggregates with a somewhat uniform shape, structure and density were selected. Experiments were deemed invalid, and their results were excluded if any of the following conditions occurred: aggregate rotation, vertical displacement of the straight micropipette, rupture of the aggregate at the onset of the test without any observed stretching or deformation, or the appearance of jumps in the stress–strain graph. These jumps in the measured force were attributed to fluctuations in suction pressure during the experiment, leading to inconsistent data. Keeping the aspirated part of the aggregate fixed typically required less suction at the beginning of the experiment compared with when the cluster was fully stretched and close to rupture. As a result, the applied suction pressure was not constant throughout the experiment and was instead adjusted to maintain a stable aspiration length. However, in some instances, the cluster was drawn further into the micropipette due to excessive suction, or it slipped out because of insufficient suction. In such cases, we discarded the ultimate tensile strength from our measurements and only reported the Young’s modulus.

#### 6.6.5. Analysis of measurements

MATLAB was used to determine the initial dimensions of the aggregate by measuring the distance between designated points in the experimental frames. In particular, the initial radius ( $R_{0}$ ) and length ( $L_{0}$ ) of the aggregate were determined from the first frame. The initial length was measured as the distance between the openings of the two micropipettes. To ensure measurement accuracy, each measurement was repeated five times across different positions of the cluster, with the mean and standard deviation calculated and reported for subsequent calculations. The error bars in figure 2c are error propagations based on the formula used to calculate the stress–strain relationship using these standard deviations for $R_{0}$ and $L_{0}$ as well as the standard deviation for $k_{p}$ (from several calibration measurements on the same micropipette). The errors for the stress are so small that they are not visible in the graph. We also used MATLAB to determine the deflection of the L-shaped micropipette as a function of time, x(t) . This deflection was used to calculate the applied force as a function of time, F(t) . Additionally, the change in the length of the cluster as $\Delta L=x_{s}-x$ , where $x_{s}=vt$ is the motion of the straight micropipette. The Young’s modulus was determined by fitting a line to the first 10−30 data points in the stress–strain graph.

#### 6.6.6. Validation and replication

To ensure consistency and accuracy, stiffness measurements were performed on 5 to 25 clusters from each prepared cell media sample.

### 6.7. Statistical analysis

We performed Welch’s *t*‐test (MATLAB ‘ttest2’) to statistically validate the difference between (i) unactivated and activated T-cells with respect to expression levels of CD69 and CD25 (figure 1c–d) and (ii) Young’s moduli of aggregates activated by different agents (figure 7).

## Data Availability

The datasets used for plotting all graphs in the paper and examples of raw data files are shared on Zenodo [78]. Supplementary material is available online [79].
